# Beyond Ischemia: A Curious Case of Electrocardiographic Abnormalities in COVID-19 Without Myocardial Injury

**DOI:** 10.7759/cureus.92528

**Published:** 2025-09-17

**Authors:** Sai Karthik Kommineni, Aditya Thakkar, Huthaifah Aburumman, Varshitha Kondapaneni, Michael S Donovan

**Affiliations:** 1 Cardiovascular Disease, East Tennessee State University Quillen College of Medicine, Johnson City, USA; 2 Internal Medicine, East Tennessee State University Quillen College of Medicine, Johnson City, USA; 3 Cardiovascular Disease, James H. Quillen Veterans Affairs Medical Center, Johnson City, USA

**Keywords:** autonomic nervous system dysfunction, covid-19, electrocardiography (ecg), postural orthostatic hypotension, qt interval prolongation, t-wave inversions

## Abstract

T-wave inversion (TWI) on electrocardiography (ECG) is a nonspecific finding that may reflect a wide range of cardiac and non-cardiac conditions. Since the emergence of COVID-19, there has been growing recognition of its diverse cardiovascular manifestations, including ECG abnormalities in the absence of traditional causes such as coronary artery disease. We present a case of significant TWI and QTc prolongation in a patient with COVID-19, underscoring the potential impact of the virus on cardiac electrophysiology and autonomic regulation.

A 68-year-old woman with a history of paroxysmal atrial fibrillation presented with dizziness and hypotension. ECG revealed marked TWI and QTc prolongation, despite normal troponin levels. She tested positive for COVID-19, though her course did not require respiratory support. Further evaluation with echocardiography and coronary angiography excluded ischemic or structural heart disease, and alternative non-cardiac metabolic causes of TWI were also excluded. The findings were, therefore, attributed to autonomic dysfunction in the setting of COVID-19 infection. Her symptoms and ECG changes resolved over six months, consistent with a transient process.

This case highlights the variable cardiac manifestations associated with COVID-19 and emphasizes the importance of recognizing TWI as a possible indicator of viral-induced cardiac involvement, even in the absence of conventional cardiovascular disease. It also reinforces the need for careful interpretation of ECG abnormalities in this population, given their potential prognostic significance. The transient course observed here suggests a reversible mechanism, possibly related to autonomic imbalance or direct viral effects on myocardial conduction. Further investigation is warranted to better delineate these mechanisms and inform targeted management strategies for patients with COVID-19-related cardiac involvement.

## Introduction

T-wave inversion (TWI) on electrocardiography (ECG) is a nonspecific but clinically important finding that may occur in various conditions, including myocardial ischemia, myocarditis, and electrolyte disturbances. With the emergence of COVID-19, however, new considerations have arisen in the interpretation of these ECG changes. Several studies have reported an association between COVID-19 infection and cardiovascular abnormalities, including TWI, in the absence of conventional causes such as coronary artery disease. Notably, Romero et al. (2020) demonstrated that TWI in patients with COVID-19 was associated with increased mortality and greater need for mechanical ventilation, underscoring its potential prognostic significance [1]. Similarly, Manzur-Sandoval et al. (2020) described marked TWI mimicking stress-induced cardiomyopathy, later attributed to the effects of COVID-19 [2].

Barman et al. (2020) further reported that TWI is more frequently observed in patients with severe COVID-19, highlighting the importance of careful evaluation of ECG abnormalities in this context [3]. Proposed mechanisms include direct viral injury to the myocardium, systemic inflammation, and autonomic nervous system dysregulation. We present the case of a 68-year-old woman with COVID-19 who developed significant TWI and QT interval prolongation despite the absence of structural or ischemic heart disease. This case underscores the broad spectrum of cardiac involvement in COVID-19 and illustrates the potential role of autonomic dysfunction in driving transient ECG abnormalities.

## Case presentation

​​A 68-year-old woman with a medical history significant for paroxysmal atrial fibrillation, type 2 diabetes mellitus, hypertension, and hyperlipidemia presented to the hospital due to dizziness and low blood pressure experienced at work, with a recorded reading of 79/44 mmHg. In the emergency department, the patient exhibited orthostatic hypotension, with blood pressure readings of 119/63 mmHg while lying, 118/64 mmHg while sitting, and 94/59 mmHg while standing. She also reported sinus congestion, earache, and a mild cough but denied chest pain, dyspnea, palpitations, syncope, weight gain, or lower limb swelling.

**Figure 1:**
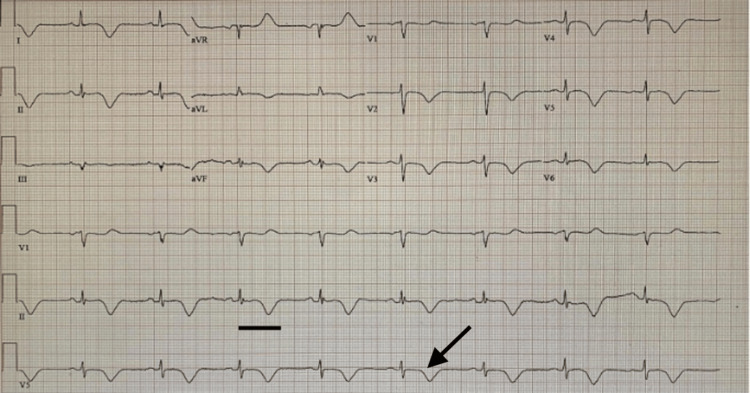
Electrocardiography shows sinus bradycardia with a heart rate of 52 beats per minute, diffuse T-wave inversions, and QT prolongation (QTc: 550 ms). The arrow represents T-wave inversions, and the black bar represents prolonged QTc measured at 550 ms.

Physical examination revealed no signs of volume overload, and her chest X-ray was normal. Her ECG showed sinus bradycardia with a heart rate of 52 beats per minute and significant T-wave changes, including diffuse TWIs and QT prolongation (Figure 1).

Serial troponin assays, obtained three times at six-hour intervals, were negative. C-reactive protein (CRP) was within normal limits, and both the complete blood count and comprehensive metabolic panel were unremarkable. She tested positive for COVID-19 infection but did not require supplemental oxygen. CT of the head showed no evidence of acute intracranial pathology.

**Figure 2:**
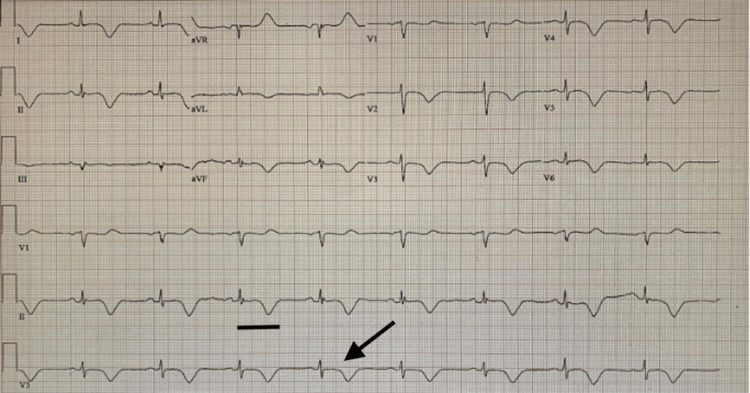
Electrocardiography shows sinus bradycardia at 50 beats per minute, diffuse T-wave inversions, and QTc prolongation (548 ms) on day three of hospital course. The arrow represents T-wave inversions, and the black bar represents prolonged QTc measured at 550 ms.

The patient’s cardiac history included a remote atrial fibrillation ablation performed five years earlier, followed by maintenance therapy with flecainide and rivaroxaban. An echocardiogram obtained two years prior demonstrated moderate concentric left ventricular hypertrophy with a preserved ejection fraction (55-60%). During the current hospitalization, serial ECGs showed persistent TWIs with QT prolongation, and orthostatic vitals remained positive. A repeat echocardiogram on hospital day two again demonstrated moderate concentric left ventricular hypertrophy, preserved ejection fraction (55-60%), and no significant valvular abnormalities. A follow-up ECG on day three continued to show persistent TWIs and QT prolongation (Figure 2).

**Figure 3:**
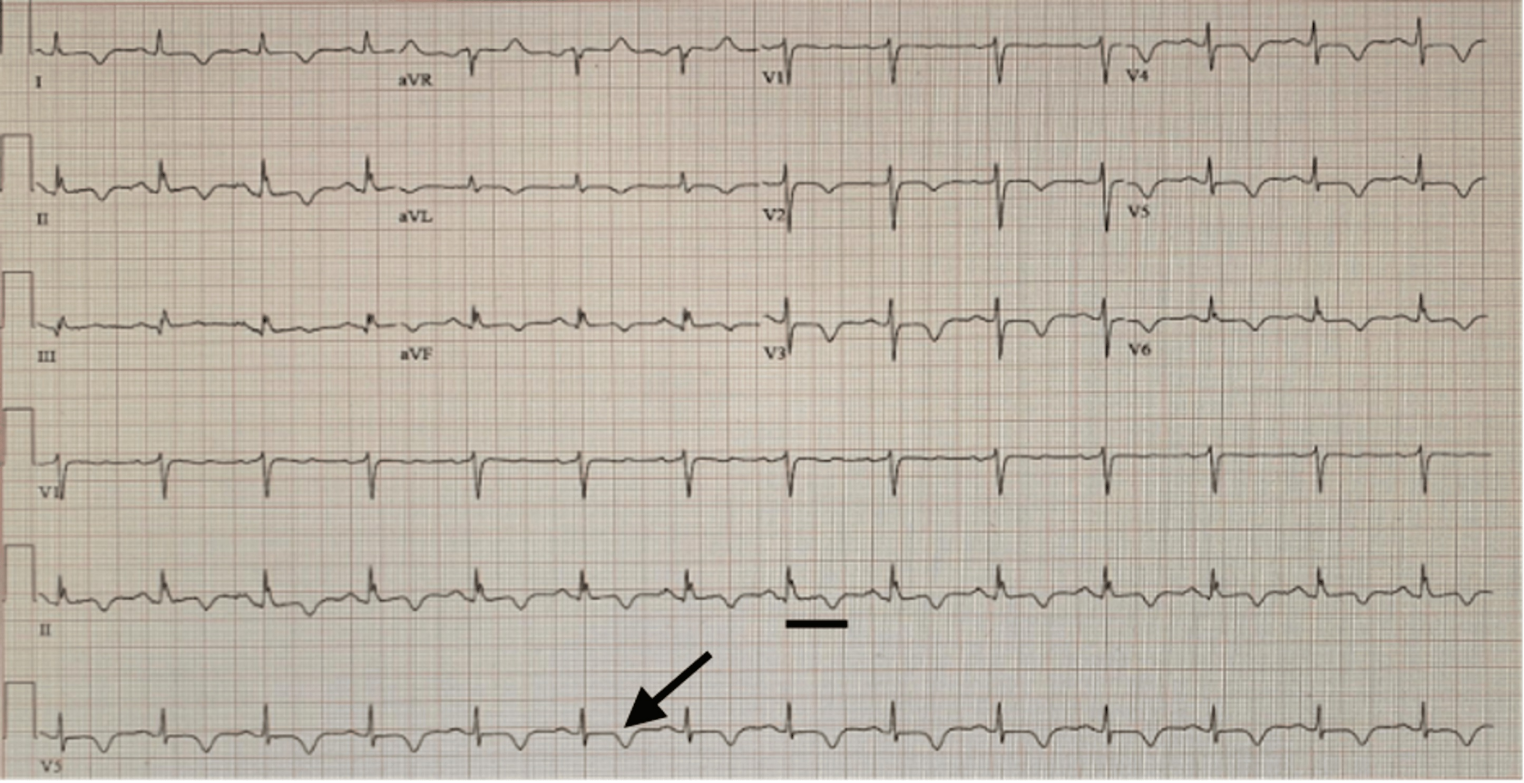
Electrocardiography shows sinus rhythm with heart rate of 84 beats per minute, persistent T-wave inversions, and improved QTc interval measured at 478 ms. The arrow represents persistent T-wave inversions, and the black bar represents improved QTc measured at 478 ms.

On hospital day four, coronary angiography was performed and revealed normal coronary arteries, effectively ruling out an ischemic etiology. The abnormal ECG changes were attributed to autonomic dysfunction secondary to COVID-19 infection. Given the persistent QT prolongation, flecainide was temporarily withheld during the index hospitalization, while anticoagulation therapy was continued. On hospital day five, the patient’s orthostatic vitals normalized, and she was discharged with plans for close monitoring, including repeat outpatient ECGs. A follow-up ECG at four weeks demonstrated persistent TWIs with improvement in the QTc interval (Figure 3). At that visit, flecainide was restarted, and the electrophysiology team felt it was an unlikely contributor to the observed ECG changes.

Cardiac MRI was not performed, as it is not available at our institution and would have required transfer to another facility. At the time of discharge, the likelihood of myocarditis was considered very low given the dramatic but isolated ECG changes, absence of symptoms, normal CRP, normal echocardiogram, and the presence of orthostatic symptoms not typically associated with myocarditis. Holter monitoring was also not pursued, as continuous inpatient telemetry and serial ECGs consistently demonstrated sinus rhythm without arrhythmias.

**Figure 4:**
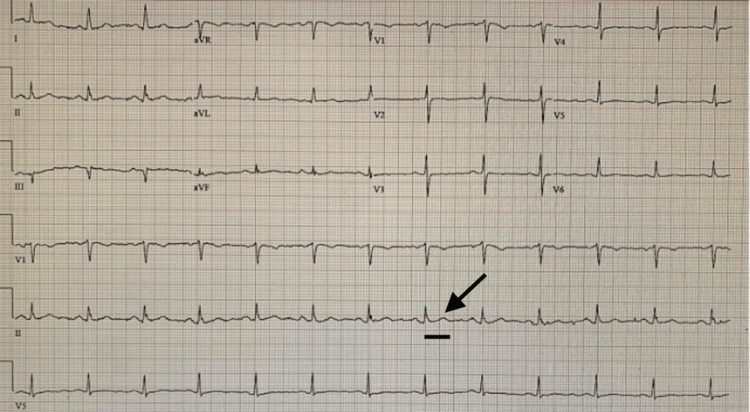
Electrocardiography shows sinus rhythm with heart rate of 78 beats per minute, upright T-waves, and improved QTc interval measured at 410 ms. The arrow represents upright T-waves, and the black bar represents improved QTc measured at 410 ms.

The patient was again followed up in the clinic six months after discharge, remained asymptomatic, and demonstrated complete resolution of TWIs with normalization of the QT interval (Figure 4). She was continued on flecainide and anticoagulation with rivaroxaban. The absence of recurrent symptoms, normalization of ECG, and preserved cardiac function on follow-up further supported autonomic dysfunction as the likely etiology.

## Discussion

Cardiac involvement in COVID-19 infection has been well-reported; it can present as acute myocardial ischemia, myocarditis, or cardiogenic shock among other less common presentations [1,2,4,5]. Some studies have estimated that the prevalence of cardiac compromise in COVID-19 infection is 20-40% [3,6]. ECG changes without and with cardiac involvement have also been described in COVID-19 infections. T-wave changes were common, with a prevalence of 48.3% in one cohort study [4]. Interestingly, ECG changes were also noticed in animals infected with the coronavirus [5,7]. TWI is a frequently encountered finding in an ECG that can be a normal variant, especially in children and younger adults, as in the case of a persistent juvenile T-wave pattern, or due to an underlying cardiac disease, such as myocardial ischemia, stress-induced cardiomyopathy, myocarditis, bundle branch block, or hypertrophic cardiomyopathy [6-9]. TWI in COVID-19 patients can carry important implications; in one case series, the mortality rate was 35% in patients with diffuse TWI, 52% in patients with elevated troponin, and 80% in patients with both [8].

The exact mechanism of cardiac damage in COVID-19 is still under ongoing investigation. Some studies have suggested direct mechanisms, and others have suggested indirect mechanisms. The evidence of the direct damage was supported by the findings of focal myocyte necrosis and interstitial edema in a rabbit model [5,7], and an endomyocardial biopsy that was performed on a patient with severe COVID-19 infection and cardiogenic shock, which showed endocardial inflammation [9,10]. One study found that 27% of patients with acute myocarditis had TWI. Moreover, the TWI correlated with areas of transmural edema on cardiac MRI (CMR), which suggests an underlying myocardial injury as a cause of these ECG changes [11].

CMR was not performed, as it is not available at our institution and would have required transfer to another facility. Clinically, it was not felt to be necessary at the time of hospitalization, as the patient remained asymptomatic with normal inflammatory markers, a normal echocardiogram, and orthostatic symptoms not typically associated with myocarditis. While this approach was guided by clinical judgment, we recognize that the absence of CMR introduces some diagnostic uncertainty.

Interestingly, our patient presented with orthostatic hypotension, but she was euvolemic, which indicates probable autonomic nervous system (ANS) involvement. ANS dysfunction has been described in COVID-19, and many mechanisms have been suggested to explain this phenomenon, some of which are very high levels of catecholamines leading to paradoxical vasodilation, withdrawal of sympathetic activity, and activation of the vagus nerve, resulting in orthostatic hypotension, and, in more severe cases, syncope [10,12].

Although patients with TWI and COVID-19 in prior case studies had an unfavorable outcome, our patient remained asymptomatic and her orthostatic hypotension resolved after a few days; further, the ECG changes resolved on subsequent outpatient follow-up. No discrete guidelines are available for the optimal management of myocardial injury in COVID-19 patients. Our management focused on ruling out co-existing ischemic heart disease and supportive care. A better understanding of ECG patterns associated with COVID-19 infection can be cost-effective. It can help formulate a prognostic tool in the future for COVID-19 patients with cardiac involvement.

## Conclusions

This case underscores the complexity of cardiovascular manifestations in COVID-19, particularly when typical causes such as coronary artery disease and overt myocardial injury are absent. The presence of marked TWIs and QTc prolongation without troponin elevation or structural heart disease, followed by complete resolution at the sixth-month follow-up, highlights the potential role of autonomic dysfunction as a contributing mechanism. Clinicians should remain vigilant in interpreting ECG changes in COVID-19 patients, integrating careful clinical evaluation, serial ECGs, and echocardiography, while reserving advanced diagnostics for cases with higher suspicion of myocarditis or other structural disease. This case adds to the growing body of literature by documenting a favorable outcome in a patient with striking ECG abnormalities and emphasizes the importance of ongoing research to clarify mechanisms, refine prognostic tools, and guide management strategies.
